# A survey demonstrating that the procedural experience of residents in internal medicine, critical care and emergency medicine is poor: training in ultrasound is required to rectify this

**DOI:** 10.1186/s13089-021-00221-x

**Published:** 2021-04-13

**Authors:** Mamdouh Souleymane, Rajkumar Rajendram, Naveed Mahmood, Amro M. T. Ghazi, Yousuf M. S. Kharal, Arif Hussain

**Affiliations:** 1 Department of Medicine, King Abdulaziz Medical City, King Abdullah International Medical Research Center, Ministry of National Guard-Health Affairs, Riyadh, Saudi Arabia; 2 College of Medicine, King Saud Bin Abdulaziz University of Health Sciences, Riyadh, Saudi Arabia; 3 Department of Intensive Care, King Abdulaziz Medical City, King Abdullah International Medical Research Center, Ministry of National Guard-Health Affairs, Riyadh, Saudi Arabia; 4 College of Medicine, Alfaisal University, Riyadh, Saudi Arabia; 5 Department of Cardiac Sciences, King Abdulaziz Medical City, King Abdullah International Medical Research Center, Ministry of National Guard-Health Affairs, Riyadh, Saudi Arabia

**Keywords:** Ultrasound-guided procedures, Education needs assessment, Curriculum development, Internal medicine, Procedural skills training, Emergency medicine, Critical care

## Abstract

**Background:**

Training in procedural skills is often suboptimal. The aim of this study was to quantify the needs of residents in internal medicine (IM), critical care (CC), and emergency medicine (EM) for instruction in ultrasound-guided procedures.

**Methods:**

All IM, EM and CC residents (*n* = 200) at King Abdulaziz Medical City, Riyadh, Saudi Arabia, were invited to participate in a questionnaire-based survey to identify skill and experience gaps. The contribution of procedural skills to patient care (i.e. applicability) and proficiency in the sterile technique required to perform ultrasound-guided procedures were rated on Likert scales. Data on training, accreditation, and experience with and without ultrasound were collected.

**Results:**

The overall response rate was 72% (IM 91%, CC 100%, EM 40%). Although the sample reported that procedural skills were very applicable, 19% (IM *n* = 25, EM *n* = 2) had not performed any procedures. However, five residents were accredited in point-of-care ultrasound, 61% of the sample had performed ultrasound-guided procedures and 65% had used landmark techniques. Whilst more internists had performed procedures using landmark techniques, CC and EM residents had performed more ultrasound-guided procedures. Whilst CC residents had not missed any opportunities to perform procedures because supervisors were less available, EM (6) and IM (89) residents had. Whilst skill gaps were only identified in the IM residency programme, experience gaps were present in all three residency programmes. The IM residency programme had larger experience gaps than the CC and EM programmes for all procedural skills.

**Discussion:**

Residents in IM, CC and EM perceive that ultrasound-guided procedures are relevant to their practice. However, the IM residents performed fewer procedures than CC residents and EM residents at least partly because internists also lack skills in ultrasound. Training in ultrasound-guided procedures may reduce the use of landmark techniques and improve patient safety. Residents in IM, CC and EM therefore require training in ultrasound-guided procedures.

**Supplementary Information:**

The online version contains supplementary material available at 10.1186/s13089-021-00221-x.

## Background

The procedural skills training of internists, intensivists and emergency medicine (EM) physicians are suboptimal worldwide [1–3]. In this context, little is known of internal medicine (IM), critical care (CC), and EM residents’ opinions on how much these skills contribute to patient care in these specialties (i.e. the applicability of bedside procedural skills).

The Saudi Commission for Health Specialties has developed specialty-specific curricula for residency training programmes in IM, CC, and EM [4–6]. These curricula include lists of procedures which residents in IM, CC and EM should be able to perform either independently or under supervision [4–6]. The training programmes at our institution implement these curricula and aim to provide the procedural skills training required.

However, to ensure patient safety, procedures must be performed by competent practitioners [7–11]. Ultrasound guidance reduces the risk of complications of many invasive procedures [8–11]. So its use is strongly recommended in many evidence-based guidelines [8–11]. However, in our setting, besides radiologists, very few clinicians are experienced with the use of ultrasound. Thus, extensive education in ultrasound to would be required to ensure safe and effective performance of procedures [12–15].

Whilst consensus documents, curricula and pathways for non-radiologists to accredit in ultrasound-guided procedures have been developed [16–18], the uptake of accreditation in point-of-care ultrasound (POCUS) has been relatively poor [19]. Thus, the aim of this study was to quantify the requirements of IM, CC and EM residents training in the twenty-first century for instruction in procedural skills by exploring their perceived needs and quantifying their training, proficiency and experience in procedural skills.

## Methods

### Ethical approval

The institutional review board of King Abdulaziz Medical City, Riyadh (KAMC) at the King Abdullah International Medical Research Center, Riyadh, Saudi Arabia, approved this study.

### Sample size calculation

Assuming a response distribution of 50%, it was estimated that 132 responses would be required from the target population (*n* = 200; IM 85/108, CC 16/16, EM 64/76) to achieve an error margin of 5% at a confidence level of 95%.

### Participants

The academic year for residency programmes in Saudi Arabia begins on 1st October. In August 2019, the IM and EM residents training at KAMC during the academic year 2018/2019 were invited to participate. In May 2020 the CC residents training at KAMC during the academic year 2019/2020 were invited to participate. Informed consent was obtained.

### Survey development

A validated questionnaire investigating procedural skills was developed with input from the curricula for residency training in IM, CC and EM in Saudi Arabia [4–6], the literature on procedural skills training [12, 19] and the applications [7–11] and competencies for the performance of procedures with and without ultrasound [16–18, 20–22]. The initial questionnaire had five sections:

- 1. Demographic information (i.e. gender, specialty, postgraduate year of training [PGY]).
- 2. Applicability of 10 procedural skills (i.e. needs assessment). These were chosen from the Saudi Commission for Health Specialties’ curricula which include lists of procedures which residents in IM, CC and EM should be able to perform either independently or under supervision [4–6].
- 3. Experience (i.e. number of procedures performed with and without ultrasound guidance).
- 4. Training in ultrasound-guided procedures, accreditation in POCUS, and missed opportunities to perform ultrasound-guided procedures (i.e. situations when a procedure which could have been performed safely at the bedside by the participant was instead performed by a radiologist).
- 5. Self-reported proficiency in each procedure with and without ultrasound guidance.

To obtain input on length, content, and clarity, the questionnaire was pre-tested with four paediatric residents. The pre-test highlighted that the questionnaire was too long, so it was shortened in response to the feedback. At our institution IM, CC, and EM are not credentialed to perform peripherally inserted central catheters or drain superficial abscesses. Lumbar punctures are only performed by or under the supervision of, the neurologists at our institution. Arterial line placement is similar to venous cannulation, but is rarely performed by IM and EM. So, the questions on experience in these four procedures were removed from “Results” section. However, the questions on the applicability of these four procedures were retained for the needs assessment.

Whilst the performance of any invasive procedure requires a standard preparatory process, ultrasound-guided procedures also require the sterile preparation of an ultrasound probe. Thereafter, the steps required for most procedures with and without ultrasound guidance are broadly similar. So, “Strengths and limitations” section was replaced with a single question on proficiency in the sterile technique required to perform ultrasound-guided procedures (a surrogate marker of procedural proficiency). “Background”, “Methods” and “Discussion” sections were not changed.

The final version of the questionnaire (Additional file 1: Appendix 1) was reviewed by the paediatric residents who had pre-tested the first questionnaire. It was deemed acceptable and so it was administered to the residents in IM, CC and EM as a paper-based questionnaire.

### Study outcomes

Perceived applicability was assessed on a Likert scale (1 very poor, 2 poor, 3 fair, 4 good, 5 very good). Proficiency in the sterile technique required to perform ultrasound-guided procedures (i.e. a surrogate for ability) was self-reported on the same scale. Training and accreditation were determined using closed questions (i.e. Y/N). Experience was reported on an incremental scale (0, 1–2, 3–5, 6–9, ≥ 10). Missed opportunities were also reported on an incremental scale (never, a few times, many times, most of the time, not applicable). The skill gaps were determined by comparing applicability with proficiency, training and accreditation in POCUS. The experience gaps were determined by comparing applicability with procedural experience.

### Statistical analysis

Data were analysed using standard descriptive statistical techniques. The responses of the IM, CC, and EM residents were analysed separately and as a cohort. The IM residents’ responses stratified by PGY and gender are presented in Additional file 2: Appendix 2. Meaningful stratification of the responses of the CC residents and the EM residents was not possible because of the small numbers of responses from residents in specialties. Interval data described on a 5-point Likert scales were presented as both frequency and mean (± standard deviation (SD)) as described previously [12, 23, 24]. These data were compared using a *t*-test or analysis of variance (ANOVA) as appropriate to enable comparison with previous studies [12]. Categorical data were compared using a Chi-squared test if unpaired or McNemar’s test if paired. All analyses were performed using Excel version 2016 (Microsoft, USA).

## Results

### Demographics and response rates

Participants’ demographic data and the response rates are shown in Table 1. The overall response rate was high (72.0%). A total of 98 IM residents (90.7%), 16 CC residents (100%) and 30 EM residents (41.7%) participated. All 144 participants completed the questionnaire and all responses were included in the final analysis.

**Table 1:** Demographics and response rates

Grade	Specialty (N, RR %)			
	Internal medicine	Critical care	Emergency medicine	All
PGY1	31 (94%)	6 (100%)	12 (60%)	49 (83%)
PGY2	25 (89%)	6 (100%)	6 (32%)	37 (70%)
PGY3	25 (89%)	2 (100%)	7 (35%)	34 (68%)
PGY4	17 (89%)	2 (100%)	5 (29%)	24 (63%)
Overall	100 (93%)	16 (100%)	30 (39%)	144 (72%)

Data are stratified by specialty, postgraduate year of training (PGY) and gender. Data are presented as frequency and percentage. The population from which the sample was obtained included 108 internal medicine residents, 16 critical care residents and 76 emergency medicine residents. Stratified response rates are given as a percentage of the number of individuals within each stratum of the population being sampled. Data stratified by gender are presented in Additional file 2: Appendix 2: Table 5. *N* number of respondents, *PGY* postgraduate year of training, *RR* response rate

The response rates of the male (M) and female (F) CC and EM residents did not differ significantly (CC, 100%; EM M 44.9%, F 29.6%, *χ*^2^ 1.7, *P* = 0.29); but women's response rates were significantly lower than men’s in IM (M 96%, F 77.4%; *χ*^2^ 9.1836, *P* = 0.002) and in the whole cohort (M 78.8%, F 57.1%, *χ*^2^ 10.1, *P* = 0.0015). Differences between male and female responses were not statistically significant.

### Training and accreditation

Table 2 shows the sample’s training in ultrasound-guided procedures and accreditation in POCUS. Only 12 residents (8.3%) had received training in POCUS as undergraduates. Forty-six (30%) had received instruction in ultrasound-guided procedures during their residency. Whilst all CC residents and most EM residents (90%) had received postgraduate training, significantly fewer IM residents had (3%; *χ*^2^ 111.5, *P* < 0.00001). Five residents (CC *n* = 3, EM *n* = 2) had accreditation in POCUS.

**Table 2:** Physicians’ self-reported accreditation and training in POCUS as undergraduates and postgraduates

Training and accreditation	Specialty			
	Internal medicine	Critical care	Emergency medicine	All
N	98	16	30	144
Undergraduate	4 (4.1% M 3)	4 (25%; M 3)	4 (13%; M 1)	12 (8.3%; M 1)
Postgraduate	3 (3.1%; M 3)	16 (100%; M 12)	27 (90%; M 20)	46 (32%; M 35)
Accreditation	0 (0%)	3 (19% M 2)	2 (7.0% M 2)	5 (3.5% M 4)

Data are stratified by specialty. Data are presented as frequency and percentage. Data for internal medicine stratified by postgraduate year of training and gender are presented in Additional file 2: Appendix 2: Table 6. *N* number of respondents, *M* male, *PGY* postgraduate year of training, *POCUS* point-of-care ultrasound

### Applicability

**Fig. 1:**
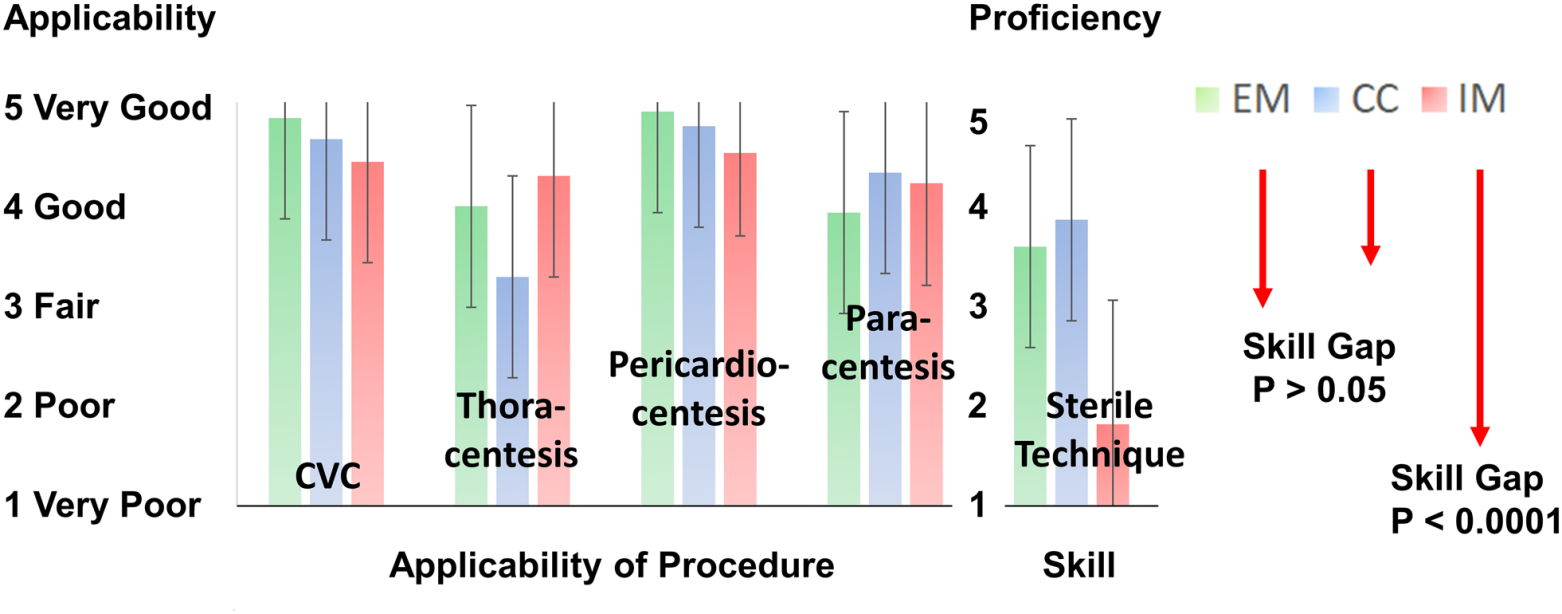
Applicability of four procedural skills, physicians’ proficiency in the sterile technique required to perform ultrasound-guided procedures, and the skill gaps. The perceived applicability of each procedure to the practice of each specialty was assessed using a Likert scale (1 very poor, 2 poor, 3 fair, 4 good, 5 very good). The proficiency in the sterile technique required to perform ultrasound-guided was self-reported on the same Likert scale. Data stratified by specialty are presented as mean ± standard deviation. The skill gap is calculated as the difference between the average applicability and the proficiency. The data from which this figure is derived are also presented in Table 3. *CC* critical care, *EM* emergency medicine, *IM* internal medicine

Of the 10 procedural skills considered in the present study, the sample reported the highest applicability for central venous catheterization (CVC) and pericardiocentesis (Table 3 and Fig. 1). The IM and EM residents considered lumbar puncture to be least relevant. The CC residents perceived that the drainage of superficial abscesses was the least relevant.

**Table 3:** Applicability of procedural skills and proficiency in the sterile technique required to perform ultrasound-guided procedures

Procedure^*^/proficiency^**^	Specialty Mean (SD)			
	Internal medicine	Critical care	Emergency medicine	All
Peripheral venous access	3.6 (1.4)	4.3 (1.3)	3.3 (1.5)	3.7 (1.4)
PICC line	4.0 (1.4)	3.6 (1.8)	3.2 (1.6)	3.6 (1.6)
CVC	4.4 (1.0)	4.9 (0.3)	4.7 (0.8)	4.7 (0.8)
Arterial line	4.0 (1.2)	4.3 (1.2)	3.5 (1.5)	3.9 (1.3)
Thoracentesis	4.3 (1.0)	4.0 (1.4)	3.3 (1.3)	3.9 (1.3)
Pericardiocentesis	4.5 (0.8)	4.9 (0.3)	4.8 (0.6)	4.8 (0.6)
Paracentesis	4.2 (1.0)	3.9 (1.4)	4.3 (1.0)	4.2 (1.1)
Arthrocentesis	3.8 (1.2)	3.9 (1.6)	3.6 (1.3)	3.5 (1.4)
Superficial abscess	3.6 (1.4)	3.3 (1.7)	3.1 (1.4)	3.4 (1.4)
Lumbar puncture	3.0 (1.4)	3.7 (1.5)	2.7 (1.4)	3.3 (1.4)
Proficiency^2^	1.9 (1.2)	3.6 (1.1)	3.9 (1.1)	3.1 (1.1)

Data are stratified by specialty. ^*^Perceived applicability of each procedure to the practice of each specialty was assessed using a Likert scale (1 very poor, 2 poor, 3 fair, 4 good, 5 very good). ^**^Proficiency in the sterile technique required to perform ultrasound-guided was self-reported on the same Likert scale. Data are presented as mean (standard deviation). Some of these data are also shown in Fig. 1. Data for internal medicine stratified by postgraduate year of training and gender are presented in Additional file 2: Appendix 2: Table 7. CVC, central venous catheter, PGY, postgraduate year of training; PICC, peripherally inserted central catheter.

### Proficiency and the skill gaps in ultrasound-guided procedures

Internal medicine residents’ self-reported proficiency in the sterile technique required to perform ultrasound-guided procedures was poor (mean 1.9 ± SD 1.2; Table 3, Fig. 1) and significantly lower than the combined mean applicability score of all procedures (3.9 ± SD 1.3, *P* < 0.00001). In contrast, CC (mean 3.6 ± SD 1.1) and EM (mean 3.9 ± SD 1.1) reported good proficiency in the sterile technique required. This was not significantly different from the combined mean applicability score of all procedures (CC mean 4.1 ± SD 1.4, *P* = 0.2; EM mean 4.0 ± SD 1.3, *P* = 0.43). These data suggest the presence of a large skill gap in IM and its absence in EM and CC (Fig. 1). However, procedural experience must also be considered.

### Procedural experience

The number of residents without any procedural experience is shown in Table 4. Whilst 27 residents (19%; IM *n* = 25, and EM *n* = 2) had not performed any procedures whatsoever, 51 (35%; IM *n* = 36, CC *n* = 6, and EM *n* = 9) had not performed a drainage procedure (i.e. thoracentesis, pericardiocentesis, paracentesis, or arthrocentesis).

**Table 4:** Residents’ procedural experience: combined procedural experience with and without ultrasound guidance

Specialty	IM (*N*, %)	CC (*N*, %)	EM (*N*, %)	All (*N*, %)
	Total (98)	Total (16)	Total (30)	Total (144)
All procedures				
None	25 (25.5%)	0 (0.0%)	2 (6.7%)	27 (19%)
US	44 (44.9%)	16 (100.0%)	28 (93.3%)	88 (61%)
Landmark	66 (67.3%)	10 (62.5%)	18 (60.0%)	94 (65%)
US and landmark	37 (37.8%)	10 (62.5%)	18 (60.0%)	65 (45%)
US only	7 (7.1%)	6 (37.5%)	10 (33.3%)	23 (16%)
Landmark only	29 (29.6%)	0 (0.0%)	0 (0.0%)	29 (20%)
Drainage procedures				
None	35 (35.7%)	6 (37.5%)	9 (30.0%)	50 (35%)
US	17 (17.3%)	9 (56.3%)	21 (70.0%)	47 (33%)
Landmark	57 (58.2%)	3 (18.8%)	10 (33.3%)	70 (49%)
US and landmark	11 (11.2%)	2 (12.5%)	10 (33.3%)	23 (16%)
US only	6 (6.1%)	7 (43.8%)	11 (36.7%)	24 (17%)
Landmark only	46 (46.9%)	1 (6.3%)	0 (0.0%)	47 (33%)

This table presents numbers of residents with any procedural experience stratified by technique (i.e. landmark techniques and ultrasound guidance). The numbers presented exceed the total number of participants because some residents had performed procedures using both landmark techniques and ultrasound guidance. The stratum entitled “Drainage procedures” includes thoracentesis, pericardiocentesis, paracentesis and arthrocentesis. The stratum entitled “All procedures” includes the drainage procedures as well as the vascular access procedures (i.e. peripheral and central venous catheterization). Data are stratified by specialty and presented as frequency. Data for internal medicine stratified by postgraduate year of training and gender are presented in Additional file 2: Appendix 2: Table 8. *CC* critical care, *EM* emergency medicine, *F* female, *IM* Internal Medicine, *L* landmark, *M* male, *N* number of respondents, *US* ultrasound, *PGY* postgraduate year of training

The number of residents with any procedural experience is also shown in Table 4. Tables 5 and 6 show the self-reported numbers of vascular access procedures (Table 5) and drainage procedures (Table 6). The procedure that the most residents had performed with ultrasound guidance was CVC (*n* = 78, 54%). Paracentesis was the procedure that the most residents had performed without ultrasound guidance (*n* = 62, 43%).

**Table 5:** Number of procedures performed: vascular access

Number of procedures	Peripheral venous access (N)						Central venous catheterization (N)					
	IM		CC		EM		IM		CC		EM	
Technique	L	U	L	U	L	U	L	U	L	U	L	U
0	71	94	9	11	22	22	73	62	8	0	17	4
1–2	12	2	1	1	3	7	10	17	4	0	7	8
3–5	8	0	2	0	0	0	14	17	1	2	2	5
6–9	6	2	2	1	4	0	0	1	0	0	1	3
≥ 10	1	0	2	3	1	1	1	1	3	14	3	10
Total	98	98	16	16	30	30	98	98	16	16	30	30

Data stratified by procedure, specialty and technique (i.e. *L* landmark, *U* ultrasound-guided) are presented as frequency. *CC* critical care, *EM* emergency medicine, *IM* internal medicine, *L* landmark, *U* ultrasound-guided

**Table 6:** Number of procedures performed: drainage procedures

Number of procedures	Thoracentesis (N)						Pericardiocentesis (N)						Paracentesis (N)						Arthrocentesis (N)					
	IM		CC		EM		IM		CC		EM		IM		CC		EM		IM		CC		EM	
Technique	L	U	L	U	L	U	L	U	L	U	L	U	L	U	L	U	L	U	L	U	L	U	L	U
0	90	93	14	10	29	29	98	95	16	16	30	30	45	85	14	8	23	11	84	96	14	12	23	18
1–2	8	4	1	4	1	1	0	1	0	0	0	0	33	11	1	7	5	8	10	2	1	4	5	10
3–5	0	0	0	0	0	0	0	2	0	0	0	0	19	1	0	1	1	5	4	0	1	0	1	1
6–9	0	0	1	1	0	0	0	0	0	0	0	0	1	0	0	0	0	2	0	0	0	0	0	1
≥ 10	0	1	0	1	0	0	0	0	0	0	0	0	0	1	1	0	1	4	0	0	0	0	1	0
Total	98	98	16	16	30	30	98	98	16	16	30	30	98	98	16	16	30	30	98	98	16	16	30	30

Data stratified by procedure, specialty, and technique (i.e. *L* landmark, *U* ultrasound-guided) are presented as frequency. Abbreviations. *CC* critical care, *EM* emergency medicine, *IM* internal medicine, *L* landmark, *N* number of respondents, *U* ultrasound-guided

Whilst fewer IM residents had performed any procedures with ultrasound guidance (*n* = 44) than without it (*n* = 66, McNemar *χ*^2^ 13.4, *P* = 0.00025), 37 had performed procedures both with and without ultrasound guidance. In contrast, all CC residents had performed ultrasound-guided procedures. Only two PGY1 EM residents (7%) had not performed any ultrasound-guided procedures. Significantly fewer CC residents (*n* = 10, 62.5%, McNemar *χ*^2^ 6.0, *P* = 0.014) and EM residents (*n* = 18, 60%, McNemar *χ*^2^ 10, *P* = 0.002) had performed procedures without ultrasound guidance.

In the whole cohort, fewer residents (*n* = 47, 33%; IM *n* = 17, CC *n* = 9, EM *n* = 21) had performed at least one drainage procedure with ultrasound guidance, than had performed at least one drainage procedure without it (*n* = 70, 49%; IM *n* = 57, CC *n* = 3, EM *n* = 10; McNemar *χ*^2^ 6.9, *P* = 0.0085). This observation was skewed by IM. More IM residents had performed drainage procedures without ultrasound guidance (McNemar *χ*^2^ 29.8; *P* < 0.00001). Fewer CC residents (McNemar *χ*^2^ 4.5, *P* = 0.034) and EM residents (McNemar *χ*^2^ 11, *P* = 0.0009) had done this.

These data suggest the presence of a large procedural experience gap in IM and imply its absence in CC and EM.

### Experience gap

Although 82 IM residents reported that the applicability of CVC was good or very good, only 44 had ever performed ultrasound-guided CVC (53.6%; *χ*^2^ 32.09; *P* < 0.00001). All CC residents and 27 EM residents described the applicability of CVC as either good or very good. All the CC and 26 EM residents had performed ultrasound-guided CVC. These observations suggest that IM has an experience gap in vascular access procedures, but CC and EM do not.

Most IM residents reported that the applicability of thoracentesis (*n* = 77), pericardiocentesis (*n* = 87), paracentesis (*n* = 75), and arthrocentesis (*n* = 63) was either good or very good, but only 19 had performed an ultrasound-guided drainage procedure. These observations suggest that internists’ experience gap in ultrasound-guided drainage procedures is even greater than that in vascular access.

The majority of CC and EM residents reported that the applicability of thoracentesis (CC *n* = 13, EM *n* = 14), pericardiocentesis (CC *n* = 16, EM *n* = 27), paracentesis (CC *n* = 11, EM *n* = 24), and arthrocentesis (CC *n* = 12, EM *n* = 13) was good or very good. Similar numbers had performed an ultrasound-guided drainage procedure (CC *n* = 9, EM *n* = 21). Whilst these data suggest that CC and EM do not have an experience gap for ultrasound-guided drainage procedures, consideration of the volume of experience is illuminating.

Tables 5 and 6 show the numbers of residents who had performed over five of each specified procedure. Whilst most CC residents (*n* = 14, 87.5%) and many EM residents (*n* = 13, 43%) had performed over five ultrasound-guided CVC, few internists had (2, 2%, *χ*^2^ 75, *P* < 0.00001). Few residents had performed over five thoracocenteses (*n* = 2), pericardiocenteses (*n* = 0), paracenteses (*n* = 8) or arthrocenteses (*n* = 1) with ultrasound guidance. Similar numbers had performed these procedures without ultrasound guidance. Thus, the majority of the sample had very little procedural experience, if any. These observations suggest the presence of large procedural experience gaps in the IM, CC, and EM residency programmes.

### Missed opportunities to perform ultrasound-guided procedures

One hundred and nine residents (76%; IM *n* = 89, EM *n* = 20) reported that they had missed opportunities to perform ultrasound-guided procedures because a supervisor was unavailable. Whilst CC residents were never in this situation, 66 residents (IM *n* = 61, EM *n* = 5) reported that this was common. Only nine IM (1 PGY1, 3%; 3 PGY2, 12%; 3 PGY3, 12%; 2 PGY4, 12%) and 10 EM (4 PGY1, 25%; 1 PGY2, 17%; 3 PGY3, 43%; 2 PGY4, 40%) residents reported that they had never missed an opportunity to perform an ultrasound-guided procedure.

Of these 19 residents, five (3.5%; IM 1 PGY1, 2 PGY2, 1 PGY4; EM 1 PGY1) reported that they had not performed any procedures despite stating that the applicability of the procedures to their practice was very good. It is unlikely that this cohort did not manage any patients who required procedures. So, this suggests that these five trainees did not want to perform procedures. The corollary of this is that the vast majority (96.5%) would like to learn practical procedures, but often miss opportunities to do them.

## Discussion

### Procedural skills and experience are poor

Whilst most of the CC and EM residents had performed procedures, alarmingly, five PGY3 (20%) IM residents and three PGY4 (17.6%) IM residents had not performed any procedures whatsoever (Additional file 2: Appendix 2: Table 8). Of the senior IM residents, only one PGY3 and three PGY4 IM residents had performed thoracentesis; a core skill for internists. As the IM and EM residents were surveyed towards the end of the academic year, the PGY4 residents had almost completed their residency training. Our observations are, therefore, representative of fellows’ technical skills at the start of their fellowships.

In 2018, Watson and colleagues [12] reported that 91, 84, and 86% of Canadian IM trainees had performed ultrasound-guided paracentesis, thoracentesis, and CVC, respectively. In the present study, significantly fewer Saudi IM residents had performed these procedures under ultrasound guidance (paracentesis 12%, thoracentesis 5%, CVC 37%; P < 0.00001). Even if all procedures performed with and without ultrasound guidance are included; fewer of our IM residents had performed procedures (paracentesis 62%, thoracentesis 12%, CVC 44%; *P* < 0.00001).

International guidelines consistently recommend the performance of procedures under ultrasound guidance. Thus, this paucity of procedural skills is likely to be a global phenomenon affecting all centres where physicians are not trained in procedural ultrasound. The skill gaps should be considered to determine whether the finite resources available for medical education should be used to rectify this.

### Skill gaps and missed opportunities for procedural skills training

Unless a physician is proficient in the generic sterile technique required to perform ultrasound-guided procedures, they cannot perform any ultrasound-guided procedure safely. Thus, the nursing staff at our institution are empowered to stop a physician performing a procedure if sterility is compromised.

The sample’s self-reported proficiency in this skill suggests the presence of a large procedural skill gap in the IM residency training programme, but its absence in CC and EM. This is probably because all CC residents and most EM residents had received postgraduate training in ultrasound-guided procedures, whilst very few internists had been trained.

The performance of ultrasound-guided procedures by IM residents without any formal postgraduate training raises governance issues and patient safety concerns. These issues must be addressed by a training programme and formal processes for supervision, governance and accreditation.

### Experience in procedures with and without ultrasound

Tables 4, 5, 6 show the sample’s self-reported procedural experience. All CC residents and most EM residents (*n* = 28) had performed ultrasound-guided procedures. Ultrasound-guided procedures are safer than landmark techniques [8–11]. However, significantly more IM residents (*n* = 66) had performed procedures without ultrasound guidance than with it (*n* = 44) and none are accredited in POCUS. In contrast, significantly fewer CC (*n* = 10) and EM (*n* = 18) residents had performed procedures using landmark techniques.

This observation suggests that training in ultrasound-guided procedures decreases the use of landmark techniques. So, instructing residents in the use of procedural ultrasound may increase patient safety. Furthermore, whilst almost all of the CC and EM residents had performed procedures, nearly 25% of IM residents had not performed any procedures. So, training in ultrasound guidance may increase the bedside performance of procedures.

### Volume of procedural experience and the experience gap

At our institution, procedural competence is not solely defined by successful performance of a minimum number of procedures under supervision. It must be determined through simulation, direct observation, and other relevant criteria outlined by the curriculum of each specialty and the residency programme directors. However, many North American residency programmes still use the historical threshold of 5 procedures to define competency [25].

Although this threshold is not used to define competence at our institution, we do believe that it provides a useful marker of residents’ exposure to procedural skills (i.e. volume of experience). As mastery of any skill requires experience, the threshold of five procedures can be used to define an experience gap.

All CC residents and most EM residents had performed over five ultrasound-guided CVC (Table 5). That only two IM residents had achieved this suggests the presence of a large experience gap in the IM residency programme in this skill. Furthermore, few residents, in any specialty, had performed over five drainage procedures with or without ultrasound (Tables 5 and 6). These observations provide evidence of large experience gaps in the performance of drainage procedures within all three residency programmes.

Our data demonstrate that training in ultrasound-guided procedures in Saudi Arabia has been variable. Whilst the residents are interested in learning this skill, IM has clearly lagged behind CC and EM. To advance, the physicians, medical educators and the regulatory bodies for medical education must fully commit to training in ultrasound-guided procedures. All stakeholders must be engaged for this endeavour to be successful. To unambiguously signal the importance of competencies in ultrasound-guided procedures, it is important that the relevant regulatory bodies make an executive decision to incorporate this into undergraduate training and reinforce its importance during postgraduate training.

Our data demonstrate that untrained residents are performing procedures with and without ultrasound guidance. Learners may erroneously believe that studies describing better outcomes with ultrasound guidance in the hands of trained operators are applicable to untrained learners [26]. However, as these skills are operator dependent [17, 18, 26], inadequate training in procedural ultrasound may increase complications [17, 18, 26].

So, healthcare systems cannot ignore the potential dangers from untrained users [18, 26]. Thus, to improve patient outcomes, there is an urgent need for qualified educators to develop a curriculum and provide appropriate training in ultrasound-guided procedures.

### Development of a curriculum and training programme for ultrasound-guided procedures

Ideally, the curriculum should include mandatory theoretical and practical training beginning with part-task trainers (i.e. simulation) [16]. If this training begins in medical school, specialty-specific training in residency and fellowship programmes could refine pre-existing skills before allowing trainees to perform procedures in patients [27]. To ensure patient safety close supervision of practice will be required.

Mastery learning is a model of competency-based training, which ensures learners achieve a learning objective before progressing to the next stage of training [28]. Given, the potential risks to patients, this is probably the most appropriate approach for procedural skills training. The Thoracentesis Assessment Tool is an example of a validated tool which can be used in a mastery learning model for procedural skills [28].

Whilst recommendations, competencies and curricula for training in ultrasound-guided procedures are available [16, 17] these must be adapted for each setting. So, every medical school and specialty must task a panel of experts with the relevant competencies to develop local curricula for ultrasound-guided procedures with clearly defined competencies and objectives.

During this process some basic principles must be followed [26]:

- 1. The curricula must be easily teachable and reliably learnable [26]. Skills must also be assessed to ensure competency and allow progression through each stage of the mastery learning process.
- 2. The use of procedural ultrasound must have clear indications (e.g. to achieve a defined goal, such as performing thoracentesis for pleural effusion).
- 3. Scopes of practice and institutional privileges must be defined [26]. Physicians must be made fully aware of their limitations [26]. When performing ultrasound-guided procedures, it is important to recognize when assistance from an expert (e.g. interventional radiologist) must be obtained.

The next challenge is implementation of the curriculum.

### Implementation of a curriculum and training programme for ultrasound-guided procedures

To facilitate this, each institution delivering the training requires champions for POCUS and ultrasound-guided procedures [26]. These individuals must ensure that regular didactic sessions are provided, appropriate equipment is available, and most importantly, hands-on training is offered [26].

To deliver this, faculty with sufficient theoretical, clinical, and practical knowledge and skills must be engaged [26]. The faculty must be fully trained, institutionally credentialed and ideally accredited [26]. These individuals must commit to training and assessing learners. Unfortunately, our data revealed that opportunities to perform procedures were missed because supervisors were not available. This observation is consistent with previous data highlighting that few general internists are able to teach procedural skills [29] and that many programmes lack trained faculty [30].

Institutional support for faculty training and the infrastructure for ongoing quality assurance processes with a secure system for archiving images must be prioritized [30]. This will require the support of the radiology department and fully certified interventional radiologists.

Curriculum implementation clearly requires substantial resources and organizational engagement. To facilitate this and ensure that important aspects are not forgotten quality metrics for medical education must be used [31]. The execution of this process in Saudi Arabia may also be guided by the previous experience of the implementation of training in ultrasound-guided procedures in other countries. Indeed, our IM residents’ perceptions of the applicability of procedural skills (Table 3, Fig. 1) were similar to those reported by IM residents training in Canada [12]. So, international standardization of basic training for ultrasound-guided procedures may be possible.

## Strengths and limitations

The accuracy of self-reported data on procedural experience can be questioned [32]. Furthermore, participants’ proficiency in the sterile technique required for ultrasound-guided procedures was investigated rather than proficiency in specific procedures. However, the reports of limited procedural experience and poor proficiency by the IM residents, and the reports of greater experience and proficiency by the residents training in CC and EM are consistent with our personal observations.

Whilst our sample perceived that the applicability of lumbar puncture was only fair, a Canadian study reported that the applicability of lumbar puncture to internists was high [12]. This difference probably reflects our institutional practices. Lumbar punctures are usually performed by neurologists at our institution. This observation highlights the critical importance of performing local needs assessments during curriculum development. Indeed, the survey instrument described in the present study could be replicated to conduct needs assessments in other centres worldwide.

Critical care residents were surveyed 7 months after the IM and EM residents. However, this is unlikely to have affected the conclusions drawn from the data as responses were received from PGY1 to PGY4 residents in all three specialties. Furthermore, the overall response rate and that of IM and CC residents was excellent. So, the desired margin of error and level of confidence were achieved. Although the EM residents’ response rate was not as good, 93.3% of EM residents had performed ultrasound-guided procedures. Thus, considering the response distribution of 93.3%, the number of responses from the EM residents was sufficient to achieve a 7% error margin at a confidence level of 83%.

The study was performed in only one hospital, so generalizability may be limited. However, international guidelines strongly recommend the performance of procedures under ultrasound guidance [8–11]. Furthermore, the IM, CC, and EM residency programmes at our institution are amongst the largest in Saudi Arabia. Our sample is therefore likely to be representative of trainees in these specialties throughout Saudi Arabia.

## Conclusion

Our study demonstrates the applicability of procedural skills to the current scope of IM, CC, and EM practice. Internists have large skill gaps in this domain whilst the CC and EM residency programmes apparently do not. However, all three specialties have experience gaps in the performance of drainage procedures. The experience gaps in IM are larger than those in EM and CC and also include CVC.

International guidelines recommend the use of ultrasound guidance rather than traditional landmark techniques. Therefore, IM, CC and EM physicians must learn ultrasound in order to perform procedures. At our institution, IM has clearly lagged behind CC and EM in this domain. Our findings are likely to reflect the situation in centres where these physicians are not trained to perform ultrasound-guided procedures. Therefore, there is an urgent need to develop curricula for training internists in procedural ultrasound. Despite regional differences in diseases, our IM residents’ responses were similar to those of Canadians. So, international standardization of training in procedural skills may be possible.

### Supplementary Information

**Additional file 1: Appendix 1.** Survey instrument.

**Additional file 2: Appendix 2.** Supplemental data tables.

## Data Availability

All authors affirm that this manuscript is an honest, accurate, and transparent account of the study being reported; that no important aspects of the study have been omitted; and that any discrepancies from the study as planned have been explained. The data that support the findings of this study are available from King Abdulaziz Medical City (KAMC), Ministry of National Guard—Health Affairs, Riyadh, Saudi Arabia. But restrictions apply to the availability of these data, which were used under license for the current study, and so are not publicly available. Data are, however, available from the authors upon reasonable request and with the permission of KAMC.

## Abbreviations

CC: Critical care
CVC: Central venous catheterization
EM: Emergency medicine
F: Female
IM: Internal medicine
KAMC: King Abdulaziz Medical City
M: Male
POCUS: Point-of-care ultrasound
PGY: Postgraduate year of training
SD: Standard deviation
