# Correction: A survey demonstrating that the procedural experience of residents in internal medicine, critical care and emergency medicine is poor: training in ultrasound is required to rectify this

**DOI:** 10.1186/s13089-023-00343-4

**Published:** 2023-11-23

**Authors:** Mamdouh Souleymane, Rajkumar Rajendram, Naveed Mahmood, Amro M. T. Ghazi, Yousuf M. S. Kharal, Arif Hussain

**Affiliations:** 1 Department of Medicine, King Abdulaziz Medical City, King Abdullah International Medical Research Center, Ministry of National Guard-Health Affairs, Riyadh, Saudi Arabia; 2 College of Medicine, King Saud Bin Abdulaziz University of Health Sciences, Riyadh, Saudi Arabia; 3 Department of Intensive Care, King Abdulaziz Medical City, King Abdullah International Medical Research Center, Ministry of National Guard-Health Affairs, Riyadh, Saudi Arabia; 4 College of Medicine, Alfaisal University, Riyadh, Saudi Arabia; 5 Department of Cardiac Sciences, King Abdulaziz Medical City, King Abdullah International Medical Research Center, Ministry of National Guard-Health Affairs, Riyadh, Saudi Arabia

**Correction: Ultrasound J (2021) 13:20** 10.1186/s13089-021-00221-x

After publication of this article [1], the authors reported that in this article the affiliation details of affiliations 1, 3 and 5 were incorrectly given as 'King Abdulaziz International Medical Research Center' but should have been 'King Abdullah International Medical Research Center'.

The original article [1] has been corrected. 
