# Supplementary material for: A survey demonstrating that the procedural experience of residents in internal medicine, critical care and emergency medicine is poor: training in ultrasound is required to rectify this
Source: Ultrasound J. 2021 Apr 13;13:20. doi: 10.1186/s13089-021-00221-x (PMC8044269; doi:10.1186/s13089-021-00221-x)
Supplement: Supplementary file 1 — Additional file 1: Appendix 1. Survey instrument. [file 13089_2021_221_MOESM1_ESM.docx]

**Survey Instrument**

**A study of the applicability of procedural skills training to**

**internal medicine, critical care and emergency medicine residents**

1. **DEMOGRAPHIC DATA**

**Specialty**

**Internal Medicine Critical Care Emergency Medicine**

**Stage of residency** PGY-1 PGY-2 PGY-3 PGY-4

**Gender** Male Female

1. **APPLICABILITY**

How applicable to your patient care are the following procedural skills?

**Rate the applicability of the procedures in the table below to your practice on a 5 point scale**

1 2 3 4 5

Very poor Poor Fair Good Very Good

| **Procedure** | **Applicability to your clinical practice?** |
| --- | --- |
| Peripheral venous access | 1 2 3 4 5 |
| Central venous catheterization | 1 2 3 4 5 |
| Arterial line Insertion | 1 2 3 4 5 |
| PICC line insertion | 1 2 3 4 5 |
| Thoracentesis | 1 2 3 4 5 |
| Pericardiocentesis | 1 2 3 4 5 |
| Paracentesis | 1 2 3 4 5 |
| Joint aspiration | 1 2 3 4 5 |
| Lumbar puncture | 1 2 3 4 5 |
| superficial abscess drainage | 1 2 3 4 5 |
| Other – please specify | 1 2 3 4 5 |

1. **EXPERIENCE**

**Please consider the procedures listed in the table below. How many of each of these procedures have you performed to date using Landmark techniques (i.e. WITHOUT ultrasound guidance)?**

| **Procedure** | **How many procedures have you performed to date WITHOUT ultrasound guidance?** | | | | |
| --- | --- | --- | --- | --- | --- |
| Peripheral venous access | 0 | 1-2 | 3-5 | 6-9 | 10 or more |
| Central venous catheterization | 0 | 1-2 | 3-5 | 6-9 | 10 or more |
| Thoracentesis | 0 | 1-2 | 3-5 | 6-9 | 10 or more |
| Pericardiocentesis | 0 | 1-2 | 3-5 | 6-9 | 10 or more |
| Paracentesis | 0 | 1-2 | 3-5 | 6-9 | 10 or more |
| Joint aspiration | 0 | 1-2 | 3-5 | 6-9 | 10 or more |

**Please consider the procedures listed in the table below. How many of each of these procedures have you performed to date USING ultrasound guidance?**

| **Procedure** | **How many procedures have you performed to date USING ultrasound guidance?** | | | | |
| --- | --- | --- | --- | --- | --- |
| Peripheral venous insertion | 0 | 1-2 | 3-5 | 6-9 | 10 or more |
| Central venous catheterization | 0 | 1-2 | 3-5 | 6-9 | 10 or more |
| Thoracentesis | 0 | 1-2 | 3-5 | 6-9 | 10 or more |
| Pericardiocentesis | 0 | 1-2 | 3-5 | 6-9 | 10 or more |
| Paracentesis | 0 | 1-2 | 3-5 | 6-9 | 10 or more |
| Joint aspiration | 0 | 1-2 | 3-5 | 6-9 | 10 or more |

1. **TRAINING IN ULTRASOUND GUIDED PROCEDURES**

**Did you receive / are you receiving formal training in ultrasound guided procedures during medical school?**

No YES Approximate Total Number of Days of training

**Did you receive / are you receiving formal training in ultrasound guided procedures during post-graduate / residency training**

No YES Approximate Total Number of Days of training

**Do you have any formal accreditation in point of care ultrasound?**

No YES If Yes - what

**How many times have you encountered a situation when you wanted to perform an ultrasound guided procedure but were not able to because of lack of a supervisor?**

Never A few times Many times Most of the time Not applicable (please specify)

1. **PROFICIENCY**

This section seeks your proficiency in a skill relevant to the performance of ultrasound guided procedures.

Please rate your own proficiency on the 5-point scale below.

1 2 3 4 5

Very poor Poor Fair Good Very Good

Sterile techniques for the performance of ultrasound-guided procedures 1 2 3 4 5

**Do you have any other comments or concerns?**
