# Supplementary material for: A survey demonstrating that the procedural experience of residents in internal medicine, critical care and emergency medicine is poor: training in ultrasound is required to rectify this
Source: Ultrasound J. 2021 Apr 13;13:20. doi: 10.1186/s13089-021-00221-x (PMC8044269; doi:10.1186/s13089-021-00221-x)
Supplement: Supplementary file 2 — Additional file 2: Appendix 2. Supplemental data tables. [file 13089_2021_221_MOESM2_ESM.docx]

**APPENDIX 2.**

**Table 5. Demographics and response rates**

| **Grade / Gender** | **Internal Medicine**  **(N, RR %)** | | **Critical Care**  **(N, RR %)** | | **Emergency Medicine**  **(N, RR %)** | | **All**  **(N, RR %)** | | | |
| --- | --- | --- | --- | --- | --- | --- | --- | --- | --- | --- |
|  | **Male** | **Female** | **Male** | **Female** | **Male** | **Female** | | **Male** | **Female** | **Total** |
| **PGY1** | 22 (100%) | 9 (81.8%) | 5 (100%) | 1 (100%) | 6 (50%) | 6 (75%) | | 33 (85%) | 16 (80%) | 49 (83%) |
| **PGY2** | 17 (100%) | 8 (72.7%) | 4 (100%) | 2 (100%) | 6 (40%) | 0 (0%) | | 27 (75%) | 10 (59%) | 37 (70%) |
| **PGY3** | 23 (92%) | 2 (66.7%) | 1 (100%) | 1 (100%) | 6 (50%) | 1 (12.5%) | | 30 (79%) | 4 (33%) | 34 (68%) |
| **PGY4** | 12 (100%) | 5 (71.4%) | 2 (100%) | 0 (100%) | 4 (40%) | 1 (14.3%) | | 18 (75%) | 6 (43%) | 24 (63%) |
| **Overall** | 74 (96%) | 26 (83.9%) | 12 (100%) | 4 (100%) | 22 (45%) | 8 (29.6%) | | 108 (79%) | 36 (57%) | 144 (72%) |

Legend to Table 5. **Demographics and response rates.** Data are stratified by specialty, postgraduate year of training (PGY) and gender. Data are presented as frequency and percentage. The population from which the sample was obtained included 108 internal medicine residents (F n=31, M n=77), 16 critical care residents (F n=4, M n=12) and 76 emergency medicine residents (F n=27, M n=49). The population from which the sample was obtained included 33 PGY1 internal medicine residents (F n=11, M n=22), 28 PGY2 internal medicine residents (F n=11, M n=17), 28 PGY3 internal medicine residents (F n=3, M n=25), 19 PGY4 internal medicine residents (F n=7, M n=12). The population from which the sample was obtained included 20 PGY1 emergency medicine residents (F n=8, M n=12), 19 PGY2 emergency medicine residents (F n=4, M n=15), 20 PGY3 emergency medicine residents (F n=8, M n=12), 17 PGY4 emergency medicine residents (F n=7, M n=10). Stratified response rates are given as a percentage of the number of individuals within each stratum of the population being sampled. The response rates of the male and female CC and EM residents did not differ significantly (CC, 100%; EM M 44.9%, F 29.6%, χ2 1.7, P=0.29); but women's response rates were significantly lower than men’s in IM (M 96%, F 77.4%; χ2 9.1836, P=0.002) and in the whole cohort (M 78.8%, F 57.1%, χ2 10.1, P=0.0015). N, number of respondents; PGY, postgraduate year of training; RR, response rate.

**Table 6. Training and accreditation**

| **Specialty** | **PGY1** | **PGY2** | **PGY3** | **PGY4** | **Male** | **Female** | **Internal Medicine** |
| --- | --- | --- | --- | --- | --- | --- | --- |
| **N** | 31 | 25 | 25 | 17 | 74 | 24 | 98 |
| **Undergraduate** | 3  (9.7%; M 2) | 0  (0%) | 1  (4%; M 1) | 0  (0%) | 3  (4.1%) | 1  (3.8%) | 4  (4.1% M 3) |
| **Postgraduate** | 0  (0%) | 1  (4%; M 1) | 2  (8%; M 2) | 0  (0%) | 3  (4.1%) | 0 | 3  (3.1%; M 3) |
| **Accreditation** | 0  (0%) | 0  (0%) | 0  (0%) | 0  (0%) | 0  (0%) | 0  (0%) | 0  (0%) |

Legend to Table 6. **Internal medicine residents training and accreditation.** Data for internal medicine are stratified by postgraduate year of training and gender. Data are presented as frequency and percentage. N, number of respondents; M, male; PGY, postgraduate year of training; POCUS, point-of-care ultrasound.

**Table 7. Applicability of procedural skills and proficiency in the sterile technique required to perform ultrasound-guided procedures**

| **Procedure^*^ / Proficiency^**^** | **Internal Medicine Strata Mean (SD)** | | | | | | **Specialty** |
| --- | --- | --- | --- | --- | --- | --- | --- |
|  | **Year of residency** | | | | **Gender** | | **Internal Medicine**  **Mean (SD)** |
|  | **PGY1** | **PGY2** | **PGY3** | **PGY4** | **Men** | **Women** |  |
| **Peripheral venous access** | 3.7 (1.2) | 3.0 (1.6) | 3.6 (1.3) | 4.5 (0.6) | 3.8 (1.3) | 3.2 (1.4) | 3.6 (1.4) |
| **PICC line** | 4.4 (1.0) | 3.5 (1.6) | 3.9 (1.1) | 4.0 (1.3) | 4.0 (1.4) | 4.0 (1.1) | 4.0 (1.4) |
| **CVC** | 4.3 (1.0) | 4.6 (0.9) | 4.2 (1.2) | 4.8 (0.4) | 4.5 (0.9) | 4.2 (1.1) | 4.4 (1.0) |
| **Arterial line** | 4.0 (1.1) | 3.9 (1.5) | 3.9 (1.3) | 4.5 (0.6) | 4.1 (1.2) | 3.8 (1.2) | 4.0 (1.2) |
| **Thoracentesis** | 4.0 (1.1) | 4.5 (0.8) | 4.2 (1.2) | 4.5 (0.7) | 4.3 (1.1) | 4.4 (0.8) | 4.3 (1.0) |
| **Pericardiocentesis** | 4.4 (0.9) | 4.6 (0.7) | 4.4 (1.0) | 4.9 (0.3) | 4.5 (0.8) | 4.6 (0.6) | 4.5 (0.8) |
| **Paracentesis** | 3.9 (1.0) | 4.4 (0.8) | 4.2 (1.0) | 4.8 (0.4) | 4.2 (1.0) | 4.3 (0.9) | 4.2 (1.0) |
| **Arthrocentesis** | 3.6 (1.3) | 3.6 (1.5) | 3.8 (1.2) | 4.2 (1.1) | 3.7 (1.2) | 4.2 (0.9) | 3.8 (1.2) |
| **Superficial abscess** | 3.7 (1.2) | 3.6 (1.5) | 2.9 (1.4) | 4.4 (1.2) | 3.7 (1.4) | 3.6 (1.3) | 3.6 (1.4) |
| **Lumbar Puncture** | 2.8 (1.3) | 2.9 (1.5) | 3.0 (1.2) | 3.5 (1.5) | 3.1 (1.4) | 2.6 (1.3) | 3.0 (1.4) |
| **Proficiency^2^** | 2.0 (1.3) | 1.8 (1.3) | 1.8 (1.2) | 1.7 (1.1) | 1.8 (1.2) | 2.0 (1.2) | 1.9 (1.2) |

Legend to Table 7. **Applicability of procedural skills to internal medicine and internists’ proficiency in the sterile technique required to perform ultrasound-guided procedures.** Data for internal medicine are stratified by postgraduate year of training and gender. ^*^Perceived applicability of each procedure to the practice of each specialty was assessed using a Likert scale (1 very poor, 2 poor, 3 fair, 4 good, 5 very good). ^**^Proficiency in the sterile technique required to perform ultrasound-guided was self-reported on the same Likert scale. Some of these data are also shown in Figure 1. Data are presented as mean (standard deviation). CVC, central venous catheter, PGY, postgraduate year of training; PICC, peripherally inserted central catheter.

**Table 8. Residents’ procedural experience: combined procedural experience with and without ultrasound guidance.**

| **Specialty** | | **Internal Medicine Strata (PGY & Gender; N)** | | | | | | **IM (N)** |
| --- | --- | --- | --- | --- | --- | --- | --- | --- |
| **PGY / Gender** | | **PGY1** | **PGY2** | **PGY3** | **PGY4** | **M** | **F** | **Total (98)** |
| **All Procedures** | None | 11 | 6 | 5 | 3 | 21 | 4 | 25 |
|  | US | 3 | 15 | 17 | 9 | 34 | 10 | 44 |
|  | Landmark | 19 | 17 | 17 | 13 | 47 | 19 | 66 |
|  | US and landmark | 2 | 13 | 14 | 8 | 28 | 9 | 37 |
|  | US only | 1 | 2 | 3 | 1 | 6 | 1 | 7 |
|  | Landmark only | 17 | 4 | 3 | 5 | 19 | 10 | 29 |
| **Drainage Procedures** | None | 13 | 10 | 8 | 4 | 27 | 8 | 35 |
|  | US | 3 | 5 | 4 | 5 | 13 | 4 | 17 |
|  | Landmark | 17 | 13 | 15 | 12 | 42 | 15 | 57 |
|  | US and landmark | 2 | 3 | 2 | 4 | 8 | 3 | 11 |
|  | US only | 1 | 2 | 2 | 1 | 5 | 1 | 6 |
|  | Landmark only | 15 | 10 | 13 | 8 | 34 | 12 | 46 |

Legend to Table 8. **Internal medicine** **residents’ procedural experience: combined procedural experience with and without ultrasound guidance.** This table presents numbers of residents with any procedural experience stratified by technique (i.e. landmark techniques and ultrasound guidance). The numbers presented exceed the total number of participants because some residents had performed procedures using both landmark techniques and ultrasound guidance. The stratum entitled "Drainage procedures" includes thoracentesis, pericardiocentesis, paracentesis and arthrocentesis. The stratum entitled "All procedures" includes the drainage procedures as well as the vascular access procedures (i.e., peripheral and central venous catheterization). Data are stratified by specialty. Data are presented as frequency. CC, critical care; EM, emergency medicine; F, female; IM, Internal Medicine; L, landmark; M, male; N, number of respondents; US, ultrasound; PGY, postgraduate year of training.

**Table 9. Numbers of residents with any experience in specific procedures and no procedural experience**

| **Specialty** | **IM (N)** | | **CC (N)** | | **EM (N)** | | **All (N)** | |
| --- | --- | --- | --- | --- | --- | --- | --- | --- |
|  | **Total (98)** | | **Total (16)** | | **Total (30)** | | **Total (144)** | |
| **Technique** | **L** | **U** | **L** | **U** | **L** | **U** | **L** | **U** |
| **CVC** | 25 | 36 | 8 | 16 | 13 | 26 | 46 (32%) | 78 (54%) |
| **Thoracentesis** | 8 | 5 | 2 | 6 | 1 | 1 | 11 (8%) | 12 (8%) |
| **Pericardiocentesis** | 0 | 3 | 0 | 0 | 0 | 0 | 0 | 3 (2%) |
| **Paracentesis** | 53 | 13 | 2 | 8 | 7 | 19 | 62 (43%) | 40 (28%) |
| **No procedures** | 32 | 54 | 6 | 0 | 12 | 2 | 50 (35%) | 56 (39%) |
| **No drains** | 42 | 81 | 13 | 7 | 20 | 9 | 75 (52%) | 97 (67%) |

Legend to Table 9. **Numbers of residents with any experience in specific procedures and no procedural experience** Data are stratified by specialty and are presented as frequency. CVC, central venous catheter; N, number of respondents; U, ultrasound-guided; L, landmark; PGY, postgraduate year of training. Drains includes thoracentesis, pericardiocentesis, paracentesis and arthrocentesis.

**Table 10. Numbers of residents who had performed more than five of each specified procedure**

| **Specialty** | **IM (N)** | | **CC (N)** | | **EM (N)** | | **All (N)** | |
| --- | --- | --- | --- | --- | --- | --- | --- | --- |
|  | **All (98)** | | **All (16)** | | **All (30)** | | **Total (144)** | |
| **Technique** | **L** | **U** | **L** | **U** | **L** | **U** | **L** | **U** |
| **central line** | 1 | 2 | 3 | 14 | 4 | 13 | 8 (5.5%) | 29 (20%) |
| **Thoracentesis** | 0 | 0 | 1 | 2 | 0 | 0 | 1 (0.7%) | 2 (1.4%) |
| **Pericardiocentesis** | 0 | 0 | 0 | 0 | 0 | 0 | 0 (0%) | 0 (0%) |
| **Paracentesis** | 1 | 1 | 1 | 0 | 1 | 6 | 3 (2.1%) | 7 (4.9%) |

Legend to Table 10. **Numbers of residents who had performed more than five of the specified procedures.** Data are stratified by specialty and are presented as frequency. Abbreviations. CC, critical care; EM, emergency medicine; IM, Internal Medicine; L, landmark; N, number of respondents; U, ultrasound-guided; PGY, postgraduate year of training.
